# Prevalence of Poor Sleep Quality in Perinatal and Postnatal Women: A Comprehensive Meta-Analysis of Observational Studies

**DOI:** 10.3389/fpsyt.2020.00161

**Published:** 2020-03-13

**Authors:** Yuan Yang, Wen Li, Tian-Jiao Ma, Ling Zhang, Brian J. Hall, Gabor S. Ungvari, Yu-Tao Xiang

**Affiliations:** ^1^ Unit of Psychiatry, Institute of Translational Medicine, Faculty of Health Sciences, University of Macau, Macau, China; ^2^ Center for Cognition and Brain Sciences, University of Macau, Macau, China; ^3^ Department of Psychiatry, Southern Medical University Nanfang Hospital, Guangdong-Hong Kong-Macao Greater Bay Area Center for Brain Science and Brain-Inspired Intelligence, Guangdong, China; ^4^ Department of Social Medicine and Health Management, School of Public Health, Jilin University, Changchun, China; ^5^ The National Clinical Research Center for Mental Disorders & Beijing Key Laboratory of Mental Disorders, Beijing Anding Hospital & the Advanced Innovation Center for Human Brain Protection, Capital Medical University, Beijing, China; ^6^ Global and Community Mental Health Research Group, Department of Psychology, University of Macau, Macau, China; ^7^ Division of Psychiatry, School of Medicine, University of Western Australia, Perth, WA, Australia; ^8^ The University of Notre Dame Australia, Fremantle, WA, Australia

**Keywords:** sleep quality, perinatal, postnatal, women, meta-analysis

## Abstract

**Background:**

Sleep disturbance is common in perinatal and postnatal women, but the epidemiology of sleep problems is highly variable in these populations. This was a meta-analysis that examined the prevalence of poor sleep quality and its correlates among perinatal and postnatal women.

**Methods:**

A systematic search of both international and Chinese databases (PubMed, EMBASE, PsycINFO, Web of Science, CNKI, and Wangfang) was performed. Studies with data on sleep quality measured by the Pittsburgh Sleep Quality Index (PSQI) were included.

**Results:**

Forty-two studies were included for analyses. The prevalence of poor sleep quality was 54.2% (95% CI: 47.9–60.5%) in perinatal and postnatal women, with 44.5% (95% CI: 37.6–51.6%) in perinatal women and 67.2% (95% CI: 57.6–75.5%) in postnatal women. The pooled total PSQI score was 7.54 ± 0.40 (95% CI: 6.75–8.33), while the average PSQI component scores varied from 0.13 ± 0.04 for use of sleeping medication to 1.51 ± 0.17 for habitual sleep efficiency. Maternal age, study site, survey year, comorbidity, PSQI cut-off value, and quality assessment score had significant moderating effects on the prevalence of poor sleep quality.

**Conclusion:**

Given the negative impact of poor sleep quality on health outcomes and well-being, regular screening for poor sleep quality and effective interventions should be conducted for this population.

## Introduction

The perinatal and the postpartum period are critical time-windows for women because of the changes in their physiology, social situation, and psychological well-being, all of which influences sleep quality (1). Sleep problems, such as poor sleep quality and sleep disturbance, often occur in perinatal and postnatal women. For instance, 14–76% of expectant mothers experience clinically significant insomnia symptoms (2, 3), while the figure increases up to 87.5% in postpartum women (4). The wide- ranging prevalence across studies are partly due to different sample demographic characteristics, sampling methods, and assessment tools. In addition, the definition of the perinatal period is inconsistent, which leads to bias due to exposure misclassification. It usually refers to the period before and after delivery, which usually begins at the 20^th^ to 28^th^ week of gestation and ends 1 to 4 weeks after childbirth (5). Some researchers even classify the perinatal period as the whole pregnancy and 1 year postpartum (6). In China, perinatal time is defined as the period that starts at the 28^th^ week of pregnancy and ends 1 week after delivery (7).

Women experience changes in sleep patterns and increased levels of tiredness after childbirth (8). For example, postnatal women usually sleep less and worse during the early days following child delivery than during pregnancy and/or other time periods of reproductive age (9). Consequently, after delivery, women often reported more daytime napping, decreased total sleep time, and poorer sleep efficiency compared with late pregnancy (10), all of which affects sleep quality. In addition, some demographic and clinical characteristics are significantly associated with poor sleep quality (11, 12). For perinatal women, advanced maternal age, fluid retention, anemia, discomfort (e.g., uncomfortable sleeping positions), and body pain, as well as mood disturbance (e.g., depressive symptoms), were correlates of sleep disturbances and quality (13–15). As for postnatal new mothers, family/social support, postpartum stress, demands from the infant (i.e., nighttime feeding and care), physical changes, and bed sharing/sleeping with the infant affects sleep quality (16–18). In addition, parity was another potential factor, as multipara women usually have less efficient sleep than nulliparas women from prepregnancy until 3 months postpartum (19). Moreover, compared to vaginal deliveries, caesarean sections were associated with poorer sleep quality and more frequent nighttime awakenings (20). Poor sleep quality could lead to negative physical and mental health outcomes (21). For example, poor sleep quality in pregnancy may increase the chances of preterm birth and longer labor (22, 23) and increase the risk of depression and suicidal ideation during pregnancy and the postnatal period (6, 24).

In order to allocate health resources and reduce the negative impact of poor sleep quality on health outcomes, it is important to understand its pattern and associated factors. Some studies have examined the prevalence of poor sleep quality, but the findings were mixed. Sedov et al. (1) conducted a meta-analysis of sleep quality during pregnancy and found that 45.7% of pregnant women experienced poor sleep quality and gestational age was a moderating factor. However, postpartum women were excluded, and only English databases were searched, restricting the generalizability of the findings.

Sleep quality, defined as an individual's subjective perception about his or her sleep (25), could be measured by both objective [e.g., polysomnography (PSG) and actigraphy (25)] and subjective methods [e.g., sleep diary and standardized instruments, such as the Pittsburgh Sleep Quality Index (PSQI)]. The PSQI is a widely used questionnaire on sleep quality in the past month (26). It has been translated and validated in many populations, such as in Chinese (27), Japanese (28), Korean (29), French (30), Kurdish (31), Portuguese (32), Serbian (33), Hungarian (34), and Persian (35). The psychometric properties of the PSQI–Chinese version is satisfactory, with the Cronbach's alpha of 0.734 (27). The PSQI total score ranges from 0 to 21, with higher scores indicating poorer sleep quality. The most commonly used cut-off value for poor sleep quality is 5 (26).

### Study aim

The aim of this meta-analysis was to examine the prevalence of poor sleep quality in perinatal and postpartum women and investigate its associated moderators. Following previous meta-analysis and empirical studies (1, 36, 37), associated moderators of poor sleep quality in perinatal and postpartum women were preidentified. In order to reduce heterogeneity caused by different measures, only studies using PSQI were included.

## Methods

### Literature Search

This meta-analysis was conducted according to the guidance of the preferred reporting items for systematic reviews and meta-analyses (PRISMA). Three investigators (YY, WL, TJM) independently and systematically conducted a literature search in PubMed, EMBASE, PsycINFO, Web of Science, CNKI, and Wangfang from their inception dates until 28th March 2019, using the following search words: (postpartum, postnatal, perinatal, maternal, Pittsburgh Sleep Quality Index, and PSQI. The PROSPERO registration number of this study is: CRD42019139366.

### Study Criteria

Following previous studies (38, 39), the perinatal period was defined as occurring from the 28^th^ week of gestation to 1 week postnatal, and the postpartum period was defined as occurring from the 2^nd^ week postnatal to 1 year. Original studies that fulfilled the following criteria were included: (1) papers published in English or Chinese; (2) cross-sectional, longitudinal, or cohort studies (only baseline data of cohort studies were extracted); (3) focusing on perinatal and/or postpartum women; (4) reporting data on sleep quality as measured by PSQI; (5) having data on PSQI score or the prevalence of poor sleep quality, or relevant data that could generate the prevalence of poor sleep quality. Articles including perinatal and/or postpartum women with severe sleep problems, such as restless legs syndrome (RLS) or obstructive sleep apnea (OSA), were excluded as their inclusion may lead to significant selection bias and an overestimation of the prevalence of poor sleep quality.

### Data Extraction and Quality Assessment

After duplicates were removed, the same three investigators independently screened all titles and abstracts of relevant publications and then reviewed all full texts for eligibility. Relevant data were extracted using a standardized data collection sheet, such as the first author, publication year, study design, location, sample size, mean age, depressive/anxiety status, comorbidities, PSQI cut-off value, PSQI score, and prevalence of poor sleep quality. Any disagreement was discussed and resolved by a consultation with a senior investigator (YTX).

Quality assessment was conducted by the same three authors independently using Parker's quality evaluation tool for epidemiological studies (40), with six domains: definition of the target population, representativeness of the study sample, sampling method, response rate, definition of the target symptom or diagnosis, and validation of the assessment instrument.

### Statistical Analyses

Comprehensive Meta-Analysis Program 2.0 (CMA 2.0, http://www.meta-analysis.com/) was used to analyze data. Considering various sampling methods and sample size across studies, the random-effects model was utilized in all analyses. Heterogeneity was tested by the Q and *I* ^2^ statistics (an *I* ^2^ of > 50% or a *P* value of < 0.10 was considered as significant heterogeneity) (41). In order to explore potential sources of heterogeneity, we conducted subgroup, meta-regression, and sensitivity analyses according to the following variables (42): continent, study site (multicenter vs. single site), hospital type (general vs. specialized), publication year (categorized by median splitting method), PSQI cut-off, maternal age, survey year, and quality assessment score. Publication bias was assessed using funnel plot and the Begg's regression model (43). Statistical significance in this study was set at *P* < 0.05 (two-tailed).

## Results

### Literature Search and Study Characteristics

**Figure 1:**
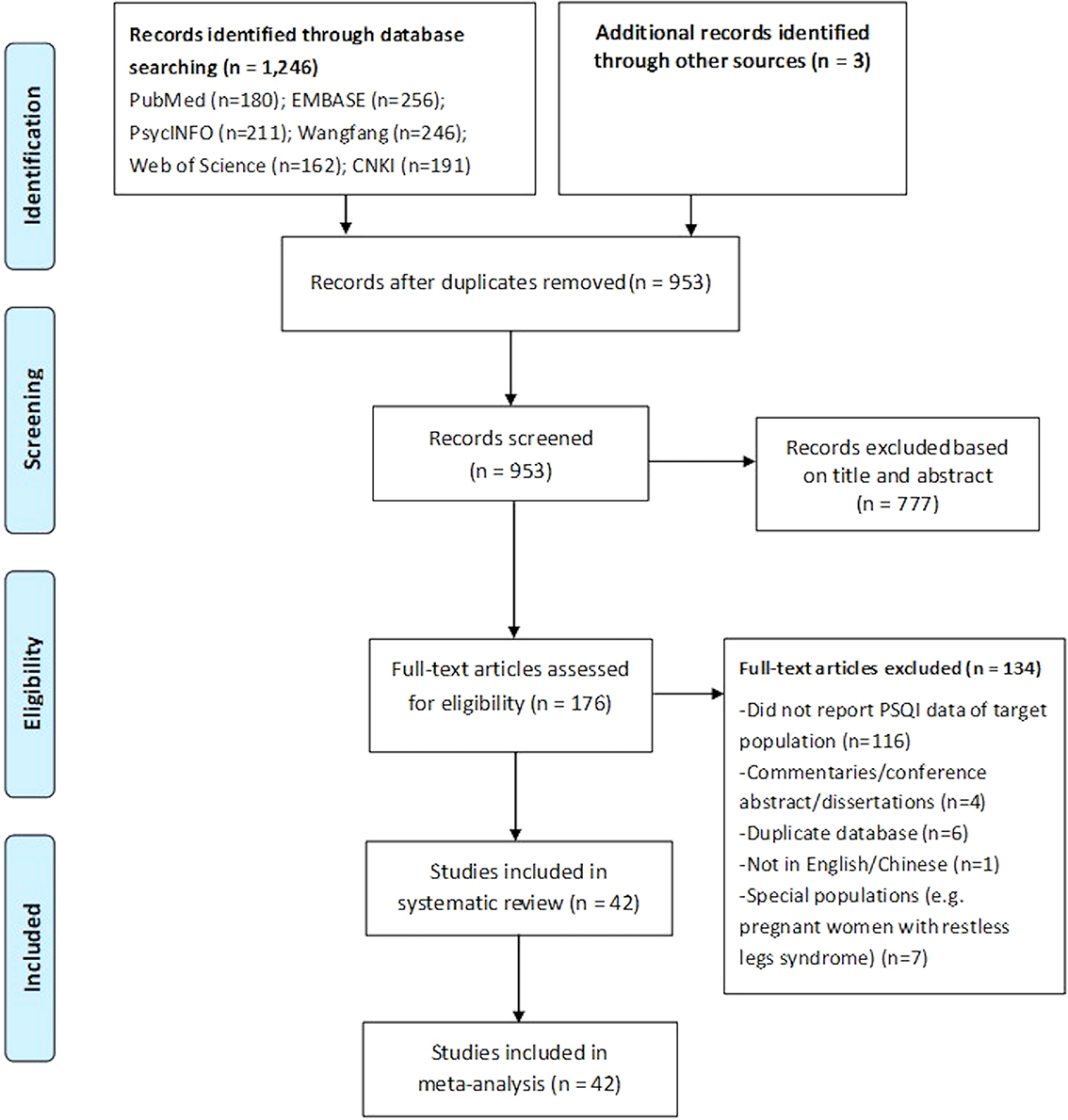
PRISMA Flowchart.

The PRISMA flowchart of literature search and selection is shown in **Figure 1**. Altogether, 1,249 relevant articles were identified. Of them, 777 were excluded by reviewing titles and abstracts. After full texts were read for eligibility, 42 studies covering 12,592 individuals were included for analyses. Of them, 28 studies reported prevalence of poor sleep quality in perinatal women, 12 reported data in postnatal women, and 2 studies included both the populations. **Table 1** displays the characteristics of the included studies. The total sample size varied from 30 to 2,830. Most of the studies were conducted in Asia (n = 36), followed by Europe (n = 4), and North America (n = 2). More than half (n = 23) of the studies were conducted in one study site, and half (n = 21) recruited patients without physical comorbidities (i.e., pregnancy hypertension, heart disease, or diabetes). Twenty studies reported data of depressive symptoms, and five reported percentage of anxiety symptoms. Seven studies utilized the Center for Epidemiologic Studies Depression Scale (CES-D) to assess participant's depressive symptoms, seven used Edinburgh Postnatal Depression Scale (EPDS), four used Self-Rating Depression Scale (SDS), while the remaining two used Hospital Anxiety and Depression Scale (HADS). Regarding anxiety symptoms, three studies utilized the Self-Rating Anxiety Scale (SAS), and two used HADS. The percentage of participants with depressive symptoms ranged from 8.4 to 57.3%, while the corresponding figure for anxiety symptoms ranged from 18.3 to 29.4%.

**Table 1:** Characteristics of the included studies.

No	First author (year)	Ref	Country	Sampling method	N	Pregnancy stage	Primipara	Physical comorbidity	Age (M ± SD)	BMI (M ± SD)	Cut-off	Depressive symptom	Anxiety symptom
1	Astuti (2017)	(44)	Indonesia	consecutive	168	Postnatal	No	No	27.5 ± 5.5	NR	≥5	NR	NR
2	Cui (2017)	(45)	China	NR	200	Perinatal	Yes	NR	30.2 ± 3.9	NR	>7	56.0	NR
3	Dorheim (2009)	(46)	Norway	consecutive	2,830	Postnatal	No	NR	30.0 ± 4.7	NR	>5	16.6	NR
4	Francis (2017)	(47)	USA	NR	640	Both	No	NR	27.0 ± 9.33	NR	≥5	NR	NR
5	Fu (2012)	(48)	China	stratified cluster	270	Perinatal	No	No	NR	NR	≥8	NR	NR
6	Gao (2012)	(49)	China	random	128	Perinatal	NR	NR	NR	NR	>7	NR	NR
7	Gunduz (2016)	(50)	Turkey	NR	92	Perinatal	NR	NR	30.4 ± 4.6	24.2 ± 3.2	>5	NR	NR
8	Hairston (2016)	(51)	Israel	NR	152	Postnatal	NR	NR	NR	NR	>5	8.4	NR
9	Han (2015)	(52)	China	NR	147	Perinatal	No	Yes	28.4 ± 4.0	NR	≥8	NR	NR
10	Huang (2004)	(53)	China (TW)	NR	163	Postnatal	Yes	No	30.0 ± 4.0	23.1 ± 2.6	≥5	50.3	NR
11	Hung (2013)	(54)	China (TW)	convenience	184	Perinatal	No	No	NR	NR	>5	NR	NR
12	Iranpour (2016)	(55)	Iran	random	353	Postnatal	No	No	27.0 ± 5.4	NR	≥5	34.8	NR
13	Ko (2012)	(56)	Korea	NR	450	Both	NR	NR	NR	NR	>5	NR	NR
14	Ko (2014)	(4)	China (TW)	NR	327	Postnatal	No	NR	30.7 ± 4.0	NR	>5	NR	NR
15	Ko (2015)	(57)	China (TW)	purposive	200	Perinatal	No	No	31.4 ± 4.0	NR	≥5	NR	NR
16	Li, Z (2018)	(58)	China	NR	288	Postnatal	NR	No	NR	NR	≥8	NR	NR
17	Li, P (2016)	(59)	China	NR	565	Perinatal	No	NR	NR	NR	≥11	25.1	NR
18	Luo (2016)	(60)	China	NR	260	Perinatal	Yes	NR	30.2 ± 3.8	NR	≥8	56.2	NR
19	Murphey (2017)	(39)	USA	purposive	33	Postnatal	Yes	No	22.3 ± 4.5	NR	>5	NR	NR
20	Naghi (2011)	(22)	Iran	consecutive	488	Perinatal	NR	NR	25.8 ± 5.7	24.1 ± 1.7	>5	NR	NR
21	Sut (2016)	(61)	Turkey	NR	152	Perinatal	No	NR	NR	NR	>5	NR	NR
22	Tobback (2017)	(62)	Belgium	NR	105	Postnatal	Yes	NR	30.4 ± 4.5	NR	>5	NR	NR
23	Tian (2018)	(63)	China	cluster	535	Perinatal	NR	Yes	28.0 ± 4.9	NR	≥7	34.2	NR
24	Tsai (2011)	(64)	China (TW)	convenience	30	Perinatal	Yes	No	30.8 ± 4.7	24.6 ± 2.2	>5	23.3	NR
25	Tsai (2016)	(65)	China (TW)	convenience	274	Perinatal	NR	NR	31.9 ± 4.0	21.1 ± 2.7	>5	23.4	NR
26	Volkovich (2016)	(66)	Israel	NR	144	Perinatal	Yes	No	29.0 ± 3.0	NR	>5	NR	NR
27	Wang, G (2018)	(67)	China	NR	262	Perinatal	NR	No	29.4 ± 3.2	26.3 ± 2.8	>5	32.9	NR
28	Wang, H (2017)	(68)	China	convenience	140	Perinatal	NR	No	29.1 ± 2.9	NR	>7	NR	NR
29	Wang, W (2017)	(69)	China	convenience	129	Perinatal	No	No	27.5 ± 4.2	NR	≥5	NR	NR
30	Wang, Y (2010)	(70)	China	NR	74	Perinatal	NR	No	26.8 ± 2.3	NR	>5	NR	NR
31	Wen, S (2018)	(14)	China (TW)	consecutive	160	Postnatal	No	No	34.4 ± 3.5	NR	≥5	NR	NR
32	Wu, X (2017)	(71)	China	NR	60	Perinatal	No	No	27.6 ± 4.1	NR	≥8	11.7	18.3
33	Wu, P (2017)	(72)	China	NR	137	Perinatal	NR	No	NR	NR	≥8	14.6	19.7
34	Yang, Y (2018)	(73)	China	NR	186	Perinatal	NR	NR	NR	NR	>5	39.9	NR
35	Yang, J (2006)	(74)	China	NR	126	Postnatal	No	No	28.5 ± 4.0	NR	>7	NR	NR
36	Zhang, W (2008)	(75)	China	NR	96	Perinatal	NR	No	27.1 ± 2.6	NR	>5	NR	NR
37	Zhang, Y (2018)	(76)	China	NR	1,000	Perinatal	Yes	NR	30.3 ± 2.9	NR	>7	50.3	NR
38	Zhang, L (2011)	(77)	China	NR	110	Perinatal	No	Yes	29.2 ± 3.6	25.9 ± 2.9	≥8	20	NR
39	Zhao, L (2018)	(78)	China	NR	200	Postnatal	No	NR	27.4 ± 3.0	NR	>7	20	23.5
40	Zhao, M (2017)	(79)	China	convenience	182	Perinatal	NR	Yes	NR	NR	>7	57.3	22.9
41	Zheng (2011)	(80)	China	random	354	Perinatal	Yes	No	28.0 ± 3.5	25.3 ± 2.4	≥8	26.7	29.4
42	Zhu (2018)	(81)	China	NR	198	Perinatal	NR	No	29.4 ± 3.1	NR	>5	17.7	NR

M, mean; SD, standard deviation; No, Number; NR, Not report; BMI, body mass index; TW, Taiwan.

### Prevalence of Poor Sleep Quality in Perinatal and Postnatal Women

**Figure 2:**
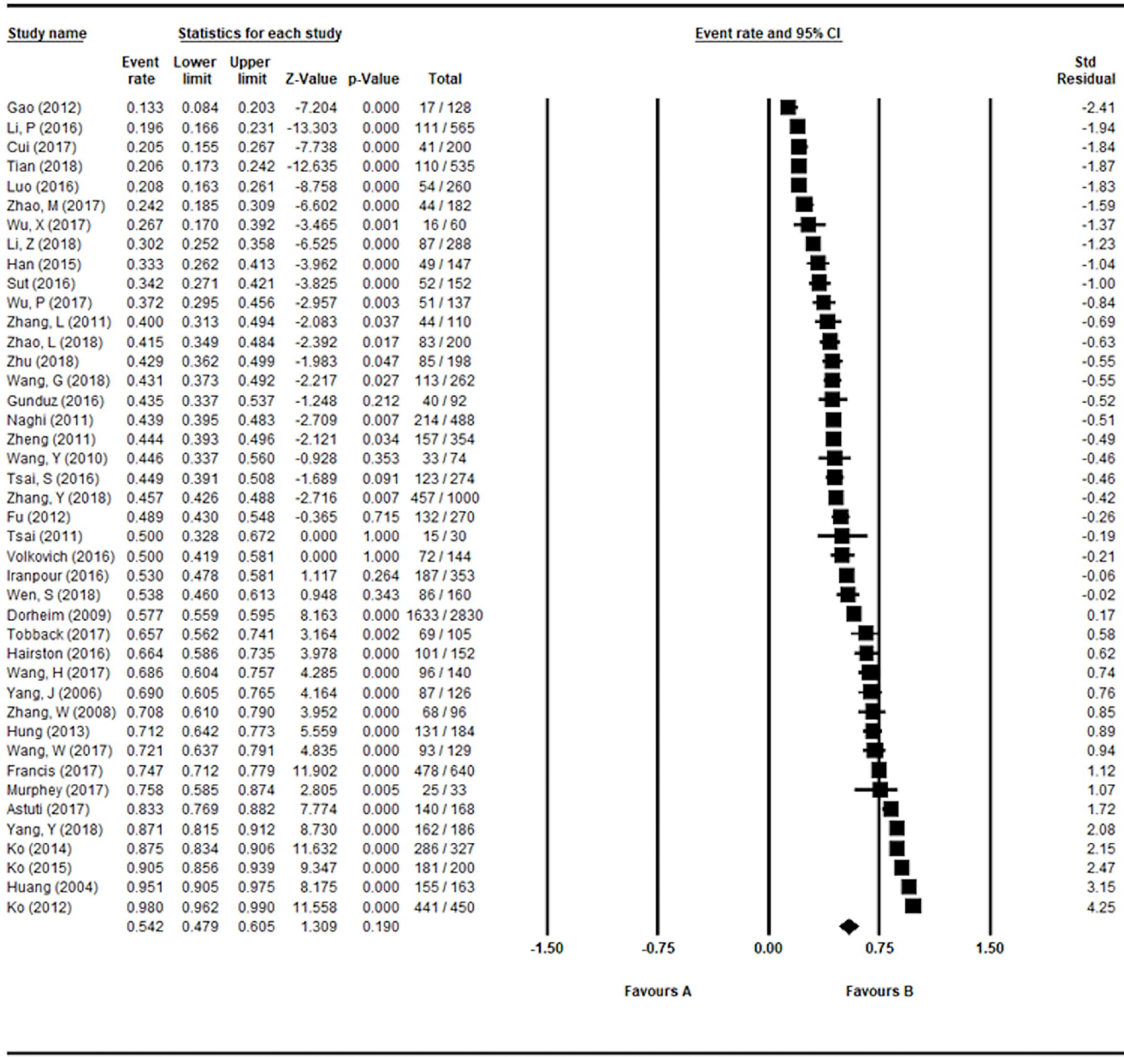
Forest plot of prevalence of poor sleep quality.

The pooled prevalence of poor sleep quality based on the 42 studies was 54.2% (95% CI: 47.9–60.5%; *I* ^2^: 97.5%) as shown in **Figure 2**, while the corresponding figure was 44.5% (95% CI: 37.6–51.6%; *I* ^2^: 96.4%) in perinatal women and 67.2% (95% CI: 57.6–75.5%; *I* ^2^: 96.5%) in postnatal women.

### The Pooled PSQI Global and Component Score

Based on the 28 studies with available data on total PSQI score and the 20 studies with PSQI component scores, the pooled PSQI total score was 7.54 ± 0.40 (95% CI of mean score: 6.75–8.33), while the average component scores of PSQI varied from 0.13 ± 0.04 (95% CI of mean score: 0.05–0.22) for use of sleeping medication to 1.51 ± 0.17 (95% CI of mean score: 0.82–1.48) for habitual sleep efficiency (**Table 2**).

**Table 2:** Pooled PSQI global and component scores.

	No. of studies	Point estimate	Standard error	Variance	95%CI
Sleep latency	20	1.23	0.11	0.01	1.00–1.45
Sleep duration	20	0.99	0.19	0.04	0.61–1.36
Sleep disturbances	20	1.38	0.06	<0.01	1.26–1.49
Daytime dysfunction	20	1.15	0.09	<0.01	0.97–1.33
Subjective sleep quality	20	1.48	0.07	<0.01	1.35–1.61
Habitual sleep efficiency	20	1.51	0.17	0.03	0.82–1.48
Use of sleeping medication	14	0.13	0.04	<0.01	0.05–0.22
Total PSQI score	28	7.54	0.40	0.16	6.75–8.33

No, Number; CI, Confidence interval; PSQI, Pittsburgh Sleep Quality Index.

### Subgroup and Meta-regression Analyses

The results of subgroup analyses are presented in **Table 3**. Postnatal women reported higher poor sleep prevalence than perinatal women (67.2% and 44.5%, respectively, P < 0.001). People in North America reported the highest prevalence of poor sleep quality (74.7%), followed by Asia (53.6%) and Europe (50.4%, P < 0.001). Studies using multicenter design showed significantly higher prevalence than those using a single site design (65.4% and 46.6%, respectively, P = 0.01). Individuals with physical comorbidities reported lower prevalence of poor sleep quality than those without (28.6% and 60.1%, respectively, P < 0.001). In addition, studies published before the year of 2016 reported significantly higher prevalence of poor sleep than those published in/after 2016 (64.7% and 47.7%, respectively, P = 0.01). Studies using lower cut-off values (i.e., ≥5) reported higher prevalence than those using high cut-offs (i.e., ≥11). Meta-regression analyses revealed that the prevalence of poor sleep quality was negatively associated with survey year (Slope = -0.079, P < 0.001), but positively associated with maternal age (Slope = 0.005, P < 0.001) and quality assessment score (Slope = 0.051, P < 0.001) (**Supplementary Figures 1–3**).

**Table 3:** Subgroup analyses of prevalence of poor sleep quality.

Subgroups	Categories (No. of studies)	Prevalence (%)	95% CI	Sample size	Events	*I* ^2^ (%)	*P* value within subgroup	Q (*P* value across subgroups)
Continents	North America (2)	74.7	71.3–77.9	673	503	0.000	0.890	37.668 **(<0.001)**
	Asia (36)	53.6	46.1–60.9	8,740	4,322	97.361	<0.001	
	Europe (4)	50.4	37.8–63.0	3,179	1,794	92.522	<0.001	
Study site	Multicenter (16)	65.4	53.7–75.5	7,616	3,746	97.904	<0.001	6.556 **(0.010)**
	Single site (23)	46.6	38.3–55.0	4,418	2,587	97.452	<0.001	
Location	Urban (31)	45.6	39.0–52.3	7,165	3,006	96.284	<0.001	9.517 **(0.009)**
	Rural (1)	44.6	33.7–56.0	74	33	–	–	
	Mixed (3)	72.7	56.8–84.3	3,638	2,251	97.851	<0.001	
Hospital type	General (24)	51.8	43.3–60.1	8,061	4,317	97.500	<0.001	5.202 (0.074)
	Mixed (6)	62.5	46.7–76.0	1,665	1,015	97.026	<0.001	
	Specialized (5)	35.8	21.8–52.7	1,730	559	97.478	<0.001	
Pregnancy	Both (2)	92.2	42.9–99.5	1,090	919	98.459	<0.001	16.414 **(<0.001)**
	Perinatal (28)	44.5	37.6–51.6	6,597	2,761	96.403	<0.001	
	Postnatal (12)	67.2	57.6–75.5	4,905	2,939	96.464	<0.001	
Primipara	Yes (9)	52.9	40.0–65.4	2,545	2,289	96.083	<0.001	0.399 (0.527)
	No (17)	58.0	48.3–67.2	8,699	6,621	97.698	<0.001	
Comorbidity	Yes (4)	28.6	20.6–38.2	1,019	974	87.158	<0.001	22.986 **(<0.001)**
	No (21)	60.1	52.1–67.5	4,408	3,569	94.912	<0.001	
BMI	Normal (5)	59.3	42.8–73.9	1,047	547	94.692	<0.001	3.459 (0.063)
	Overweight (3)	43.3	39.7–46.9	726	314	0.000	0.723	
Publication Year ^a^	Before 2016 (16)	64.7	54.8–73.5	5,977	3,643	97.252	<0.001	6.700 **(0.010)**
	In/After 2016 (26)	47.7	39.6–55.9	6,615	2,976	97.460	<0.001	
Cut-off	>5 (18)	62.9	55.0–70.2	6,077	3,663	96.088	<0.001	168.506 **(<0.001)**
	≥5 (7)	77.9	65.9–86.5	2,378	1,431	96.117	<0.001	
	>7 (15)	36.7	29.9–44.0	3,602	1,415	94.225	<0.001	
	≥7 (1)	20.6	17.3–24.2	535	110	–	–	
	≥11 (1)	19.6	16.6–23.1	565	111	–	–	

No, Number; BMI, body mass index; CI, Confidence interval; PSQI, Pittsburgh Sleep Quality Index; a: based on median split. Bolded values: <0.05.

### Study Quality Assessment, Sensitivity Analyses, and Publication Bias

The quality assessment scores ranged from 4 to 6 (**Supplementary Table 1**). Most (81%) of the studies did not utilize random or consecutive sampling method, and around half (52%) did not report response rate, or the response rate was less than 70%. Sensitivity analysis did not find individual studies that could significantly change the overall primary results. The funnel plot and Begg's tests (P = 0.06) did not find publication bias (**Supplementary Figure 4**).

## Discussion

To our best knowledge, this was the first meta-analysis to examine the prevalence of poor sleep quality in perinatal and postpartum women and investigate its associated factors. The pooled prevalence (54.2%, 95% CI: 47.9–60.5%) of this meta-analysis was similar to the findings in pregnant women (45.7%, 95% CI: 36.5–55.2%) (1), but was higher than nonpregnant populations using the same sleep assessment tool, such as college students (24.1%, 95% CI: 21.0–27.5%) (82) and older adults (35.9%, 95% CI: 30.6–41.2%) (83).

Compared to nonpregnant women, those in perinatal and postpartum period are more likely to experience acute partial sleep deprivation and chronic sleep disruption, especially during labor and the first few days after giving birth (84). Commonly reported contributing factors of poor sleep quality included level of progesterone (37), physical discomforts (85), the infant's sleep–wake patterns, and the feeding practices (86).

Subgroup analyses revealed that the prevalence of poor sleep quality was higher in postnatal (67.2%, 95% CI: 57.6–75.5%) than in perinatal women (44.5%, 95% CI: 37.6–51.6%), which confirmed some (47), but not all studies (87–89). For instance, one longitudinal study using the PSQI found that 71% of women reported poor sleep in prenatal assessment and the figure increased to 77% during postpartum period (47). However, another longitudinal study found that women's sleep quality decreased progressively from their second to third trimester, but gradually improved during postnatal period (87). Studies using actigraphy and PSG also showed that even though mothers' nighttime sleep deteriorates progressively throughout pregnancy and becomes poorest on the night before delivery, an improving trend further into the postpartum period was found (88, 89). Different study characteristics, socioeconomic contexts, and measurement tools could partly contribute to the different findings between studies.

As expected, use of lower PSQI cutoff values was associated with higher prevalence of poor sleep quality. The pooled PSQI total score in this meta-analysis was 7.54 ± 0.40 (95% CI of mean score: 6.75–8.33), which is consistent with the average PSQI score of 6.97 (95% CI of mean score: 5.30–6.85) throughout pregnancy reported previously (1). The lowest PSQI component score was 0.13 ± 0.04 (95% CI of mean score: 0.05–0.22) in the domain of “use of sleeping medication.” It is possible that pregnant and postnatal women worry about the impact of medication side effects or potential risk on their infants; therefore, they are reluctant to receive medication treatment for sleep disturbances.

Studies conducted in America and those involving multicenter and mixed locations (rural and urban) tended to report higher prevalence of poor sleep quality. This could be partly attributed to the uneven number of studies across different subgroups; for example, only two studies were conducted in America, and only three studies included participants from both urban and rural areas. Women with physical comorbidities reported lower prevalence of poor sleep than those without. It is possible that while women with comorbidities were treated for their physical complaints, they received additional care/help for their sleep problems. Meta-regression analyses revealed a decreasing trend of poor sleep quality over time. With the increased attention on sleep in the past year, women in perinatal and postnatal period could have an easier access to sleep clinics and relevant health services.

In this meta-analysis, older maternal age was associated with higher prevalence of poor sleep quality, which is consistent with most earlier findings (83, 90). Traditionally, older women tended to have heavier domestic duties and care burden, and are more likely to experience physical discomforts and slower recovery from delivery (85, 91), all of which could increase the likelihood of poor sleep quality. However, Sedov et al. (1) suggested that in pregnant women, only gestational age, but not maternal age, was related to poor sleep quality.

Several limitations should be acknowledged in this meta-analysis. First, substantial heterogeneity, which is unavoidable in epidemiological studies (92, 93), still remained although subgroup and sensitivity analyses were performed. Second, some variables that may affect sleep quality, such as depression, economic status, marital status, and interpersonal relationship difficulties, were not investigated due to insufficient data in included studies. Third, the number of studies was relatively small in some subgroups. Finally, those with severe sleep problems, such as RLS and OSA, were not included in this meta-analysis. Sleep quality should be meta-analyzed separately in these populations.

## Conclusions

In conclusion, this meta-analysis showed that poor sleep quality is common in perinatal and postnatal women. Given the negative impact of poor sleep quality on health outcomes and well-being, regular screening for poor sleep quality would be beneficial to improve sleep quality in this population.

## Data Availability Statement

The raw data supporting the conclusions of this article will be made available by the authors, without undue reservation, to any qualified researcher.

## Author Contributions

Study design: YY, YTX. Data collection, analysis, and interpretation: YY, WL, TJM, LZ. Drafting of the manuscript: YY, YTX. Critical revision of the manuscript: BH, GU. Approval of the final version for publication: all co-authors.

## Funding

The study was supported by the National Science and Technology Major Project for investigational new drug (2018ZX09201-014), the Beijing Municipal Science & Technology Commission (No. Z181100001518005), and the University of Macau (MYRG2019-00066-FHS).

## Conflict of Interest

The authors declare that the research was conducted in the absence of any commercial or financial relationships that could be construed as a potential conflict of interest.

## Abbreviations

CBTI, Cognitive behavioral therapy for insomnia; PRISMA, Preferred reporting items for systematic reviews and meta-analyses; PSG, Polysomnography; PSQI, Pittsburgh sleep quality index.
