# Supplementary material for: Prevalence of Poor Sleep Quality in Perinatal and Postnatal Women: A Comprehensive Meta-Analysis of Observational Studies
Source: Front Psychiatry. 2020 Mar 13;11:161. doi: 10.3389/fpsyt.2020.00161 (PMC7082815; doi:10.3389/fpsyt.2020.00161)

Main text: 2274 words

Summary: 199 words

Tables: 3

Figures: 2

Supplemental Table: 1

Supplemental Figure: 4

**Prevalence of poor sleep quality in perinatal and postnatal women: a comprehensive meta-analysis of** **observational studies**

Running head: sleep quality in perinatal and postnatal women

^1,2,3#^ Yuan Yang, PhD

^1,2#^ Wen Li, MD, PhD

^4#^ Tian-jiao Ma, PhD

^5#^ Ling Zhang, MD, PhD

^6,7^ Brian J. Hall, PhD

^8,9^ Gabor S. Ungvari, MD, PhD

^1,2^* Yu-Tao Xiang, MD, PhD

1. Unit of Psychiatry, Institute of Translational Medicine, Faculty of Health Sciences, University of Macau, Macao SAR;
2. Center for Cognition and Brain Sciences, University of Macau, Macao SAR;
3. Department of Psychiatry, Southern Medical University Nanfang Hospital, Guangdong-Hong Kong-Macao Greater Bay Area Center for Brian Science and Brain-Inspired Intelligence, Guangdong, China;
4. Department of Social Medicine and Health Management, School of Public Health, Jilin University, Changchun, China;
5. The National Clinical Research Center for Mental Disorders & Beijing Key Laboratory of Mental Disorders, Beijing Anding Hospital & the Advanced Innovation Center for Human Brain Protection, Capital Medical University, Beijing, China;
6. Global and Community Mental Health Research Group, Department of Psychology, University of Macau, Macao SAR;
7. Health, Behavior, and Society, Johns Hopkins Bloomberg School of Public Health, Baltimore, MD, USA;
8. Division of Psychiatry, School of Medicine, University of Western Australia, Perth, Australia;
9. The University of Notre Dame Australia, Fremantle, Australia.

^#^ These authors contributed equally to the work.

* Address correspondence to Dr. Yu-Tao Xiang, 3/F, Building E12, Faculty of Health Sciences, University of Macau, Avenida da Universidade, Taipa, Macau SAR. Fax: +853-2288-2314; Phone: +853-8822-4223; E-mail: [xyutly@gmail.com](mailto:xyutly@gmail.com;%20or).

**Supplementary table 1: Quality assessment**

|  | Targeted population was defined clearly | Random or consecutive sampling methods | Response rate was ≥ 70% | Targeted sample was representative | The definition of poor sleep quality was defined | The instruments used to assess sleep was validated | Total |
| --- | --- | --- | --- | --- | --- | --- | --- |
| 1 | 1 | 1 | 0 | 1 | 1 | 1 | 5 |
| 2 | 1 | 0 | 0 | 1 | 1 | 1 | 4 |
| 3 | 1 | 1 | 1 | 1 | 1 | 1 | 6 |
| 4 | 1 | 0 | 0 | 1 | 1 | 1 | 4 |
| 5 | 1 | 1 | 1 | 1 | 1 | 1 | 6 |
| 6 | 1 | 1 | 1 | 1 | 1 | 1 | 6 |
| 7 | 1 | 0 | 0 | 1 | 1 | 1 | 4 |
| 8 | 1 | 0 | 0 | 1 | 1 | 1 | 4 |
| 9 | 1 | 0 | 1 | 1 | 1 | 1 | 5 |
| 10 | 1 | 0 | 1 | 1 | 1 | 1 | 5 |
| 11 | 1 | 0 | 0 | 1 | 1 | 1 | 4 |
| 12 | 1 | 1 | 1 | 1 | 1 | 1 | 6 |
| 13 | 1 | 0 | 0 | 1 | 1 | 1 | 4 |
| 14 | 1 | 0 | 1 | 1 | 1 | 1 | 5 |
| 15 | 1 | 0 | 0 | 1 | 1 | 1 | 4 |
| 16 | 1 | 0 | 1 | 1 | 1 | 1 | 5 |
| 17 | 1 | 0 | 0 | 1 | 1 | 1 | 4 |
| 18 | 1 | 0 | 0 | 1 | 1 | 1 | 4 |
| 19 | 1 | 0 | 1 | 0 | 1 | 1 | 4 |
| 20 | 1 | 1 | 1 | 1 | 1 | 1 | 6 |
| 21 | 1 | 0 | 0 | 1 | 1 | 1 | 4 |
| 22 | 1 | 0 | 0 | 1 | 1 | 1 | 4 |
| 23 | 1 | 0 | 1 | 1 | 1 | 1 | 5 |
| 24 | 1 | 0 | 0 | 0 | 1 | 1 | 3 |
| 25 | 1 | 0 | 0 | 1 | 1 | 1 | 4 |
| 26 | 1 | 0 | 0 | 1 | 1 | 1 | 4 |
| 27 | 1 | 0 | 1 | 1 | 1 | 1 | 5 |
| 28 | 1 | 0 | 0 | 1 | 1 | 1 | 4 |
| 29 | 1 | 0 | 0 | 1 | 1 | 1 | 4 |
| 30 | 1 | 0 | 1 | 1 | 1 | 1 | 5 |
| 31 | 1 | 1 | 1 | 1 | 1 | 1 | 6 |
| 32 | 1 | 0 | 0 | 1 | 1 | 1 | 4 |
| 33 | 1 | 0 | 1 | 1 | 1 | 1 | 5 |
| 34 | 1 | 0 | 0 | 1 | 1 | 1 | 4 |
| 35 | 1 | 0 | 1 | 1 | 1 | 1 | 5 |
| 36 | 1 | 0 | 1 | 1 | 1 | 1 | 5 |
| 37 | 1 | 0 | 1 | 1 | 1 | 1 | 5 |
| 38 | 1 | 0 | 1 | 1 | 1 | 1 | 5 |
| 39 | 1 | 0 | 1 | 1 | 1 | 1 | 5 |
| 40 | 1 | 0 | 0 | 1 | 1 | 1 | 4 |
| 41 | 1 | 1 | 1 | 1 | 1 | 1 | 6 |
| 42 | 1 | 0 | 1 | 1 | 1 | 1 | 5 |

Supplementary Figure 1. Meta-regression of Survey Year on prevalence of poor sleep quality


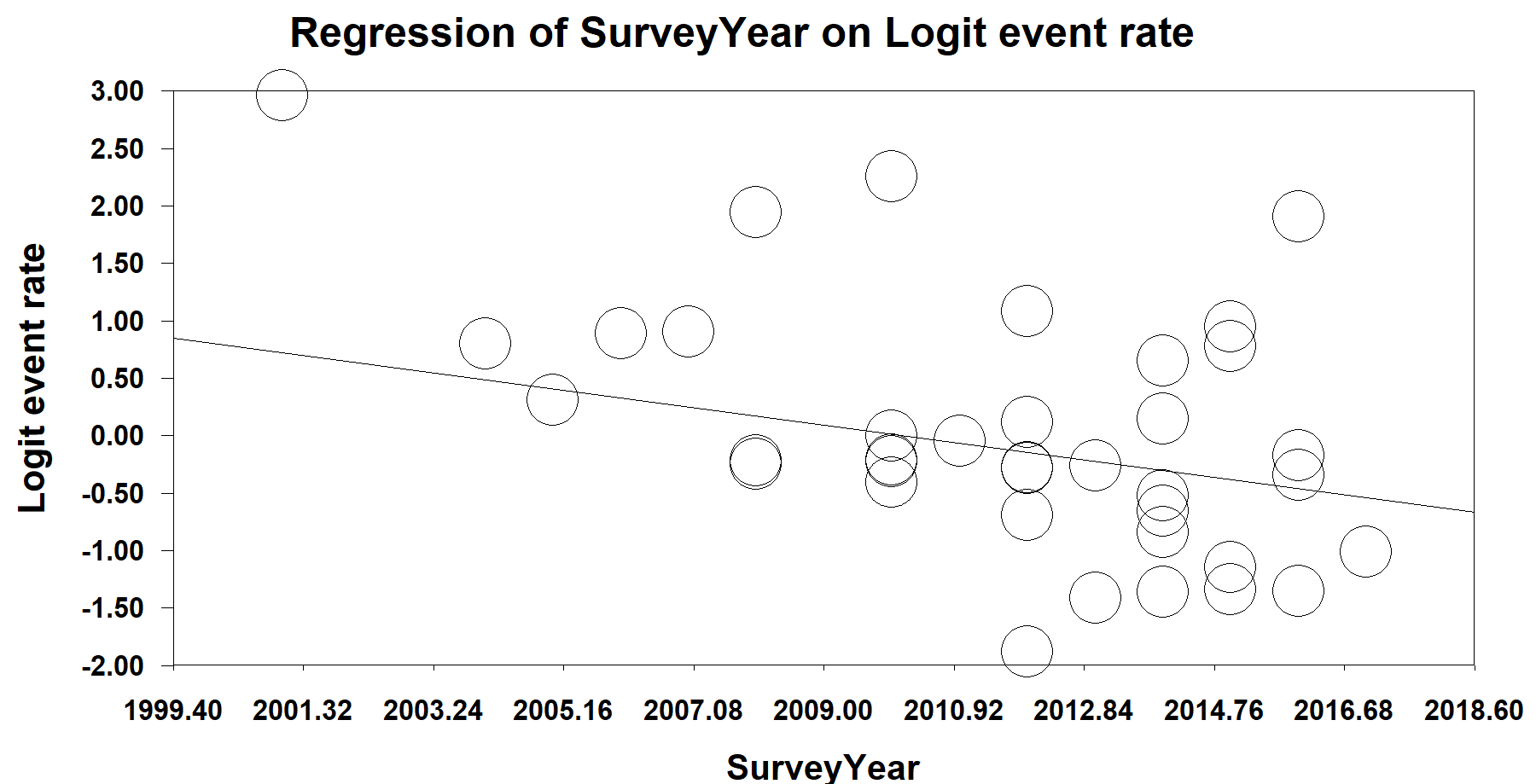


Note: Slope=-0.079, Intercept=158.811, P<0.001

Supplementary Figure 2. Meta-regression of Age on prevalence of poor sleep quality


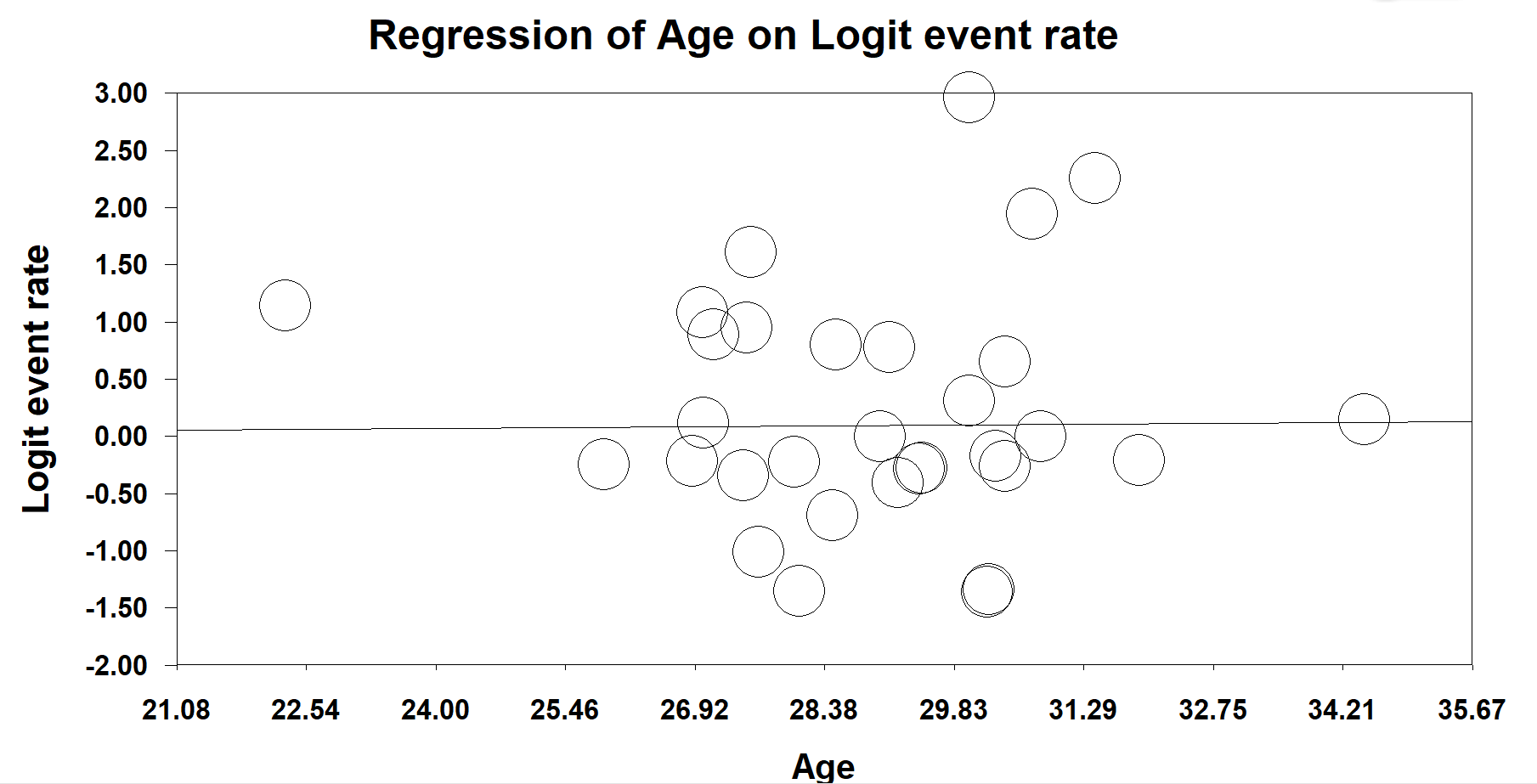


Note: Slope=0.005, Intercept=-0.052, P<0.001

Supplementary Figure 3. Meta-regression of Survey Year on prevalence of poor sleep quality


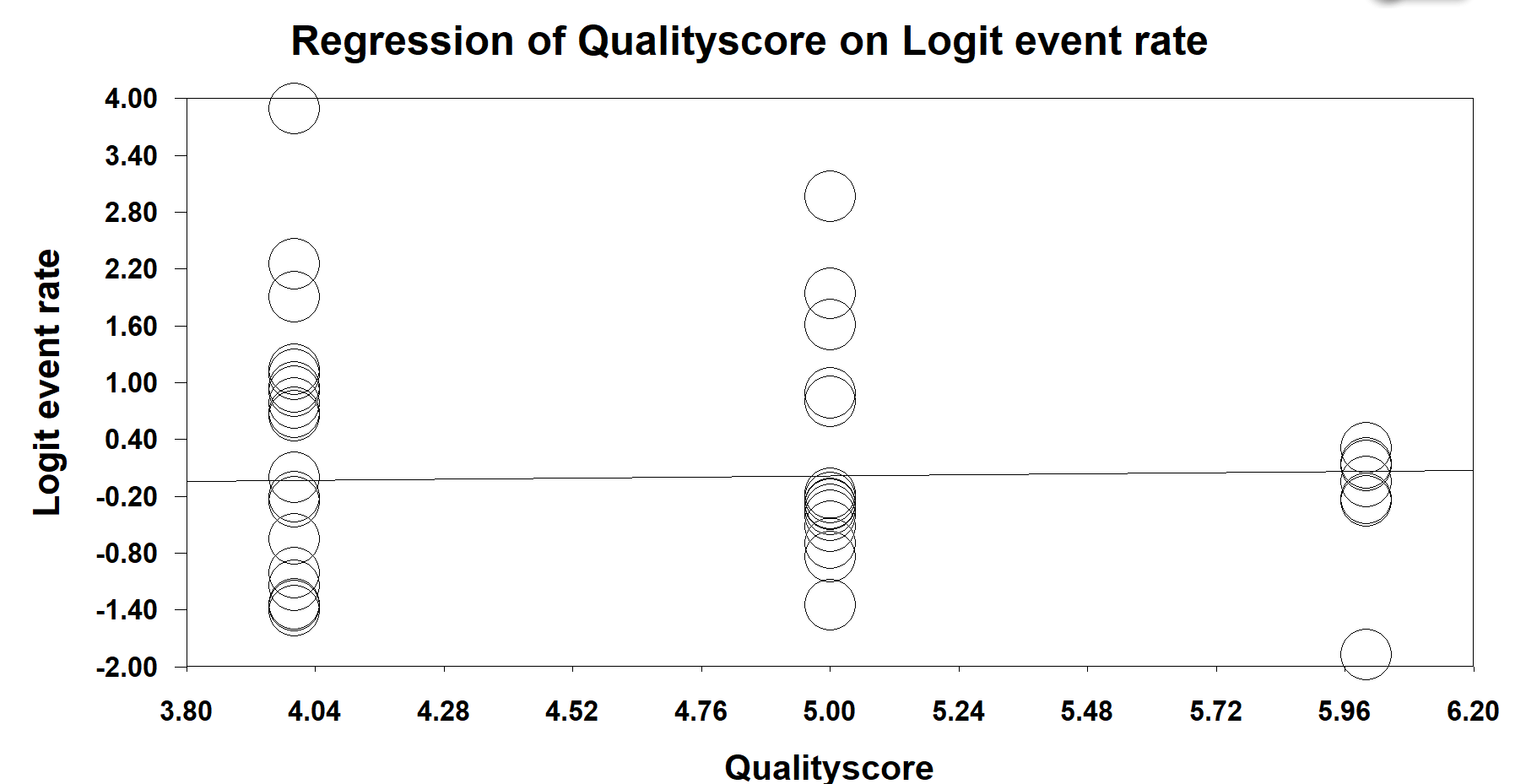


Note: Slope=0.051, Intercept=-0.241, P<0.001

Supplementary Figure 4. Funnel Plot of included studies


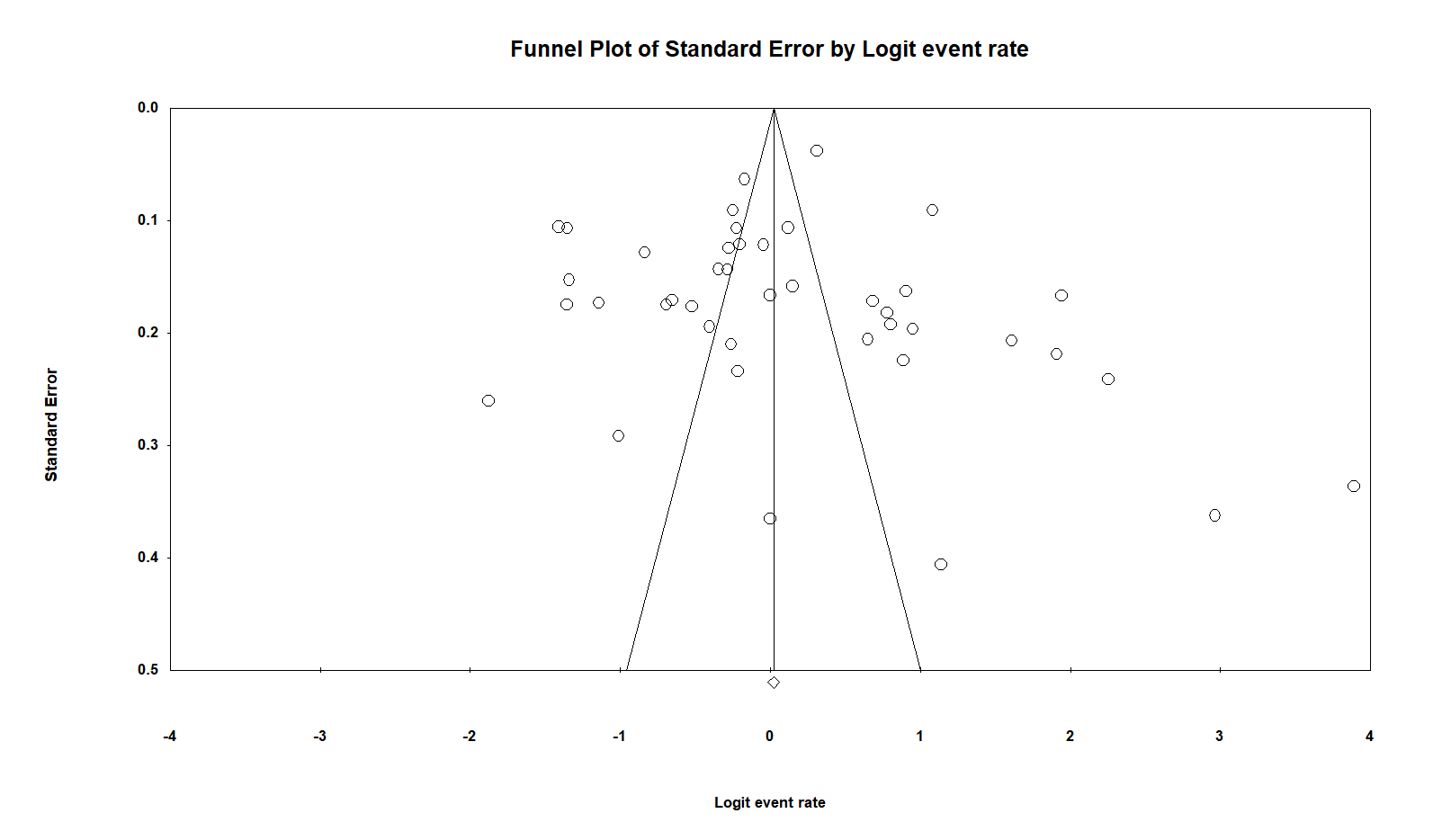

Supplement: Supplementary file 1 [file DataSheet_1.docx]
